# Bonobos innovate, plan, and transmit tool use to solve an extractive foraging task

**DOI:** 10.1016/j.isci.2026.117443

**Published:** 2026-09-04

**Authors:** Lara Zanutto, Jake A. Funkhouser, Erik P. Willems, Ava Moser, Crickette Sanz, Zanna Clay, Kathelijne Koops

**Affiliations:** 1 Ape Behaviour and Ecology Group, Department of Evolutionary Anthropology, University of Zurich, Zurich, Switzerland; 2 Department of Anthropology, Washington University in St. Louis, St. Louis, MO, USA; 3 Lester E. Fisher Center for the Study and Conservation of Apes, Lincoln Park Zoo, Chicago, IL, USA; 4 Congo Program, Wildlife Conservation Society, Brazzaville, Republic of the Congo; 5 Department of Psychology, Durham University, Durham, UK

**Keywords:** bonobos, tool use, extractive foraging, rearing history

## Abstract

The evolutionary origins of tool use remain poorly understood. Drawing inferences from living apes is challenging due to striking differences between chimpanzees (*Pan troglodytes*) and bonobos (*P. paniscus*), our two closest living relatives. Chimpanzees routinely use tools in both the wild and captivity. Conversely, wild bonobos rarely use tools, whereas captive bonobos show diverse tool use. We examined the individual (i.e., age, sex, and rearing history), social (i.e., past and present exposure to conspecifics using tools), and environmental (i.e., tool availability) tool-use drivers in sanctuary-living bonobos using a novel group-based extractive foraging task. Tool use was more frequent in adult females than in males, in human-reared than in mother-reared immatures, after greater exposure to tool use, and when tools were provided. Hence, tool use in sanctuary-living bonobos is driven by social learning opportunities and developmental biases, providing insight into the conditions that may have favored the emergence of tool use during hominin evolution.

## Highlights

• Tool use rates increased with social learning opportunities and tool availability
• Participation in the foraging task was linked to socially mediated access
• Adult female bonobos planned tool use and used tools more often than males
• Human-reared immature bonobos used tools more often than mother-reared immatures

## Graphical abstract

**Figure:**
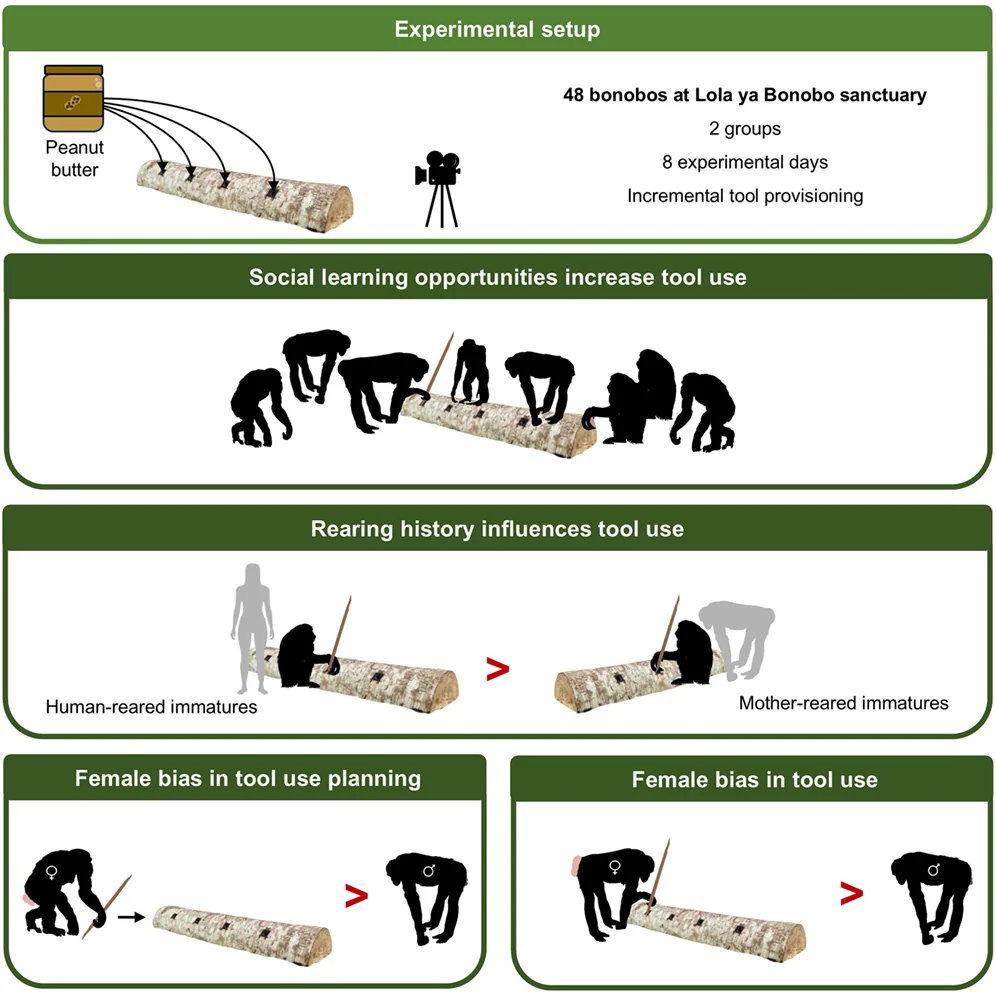

## Teaser

Wildlife behavior; Natural sciences; Zoology

## Introduction

The ability to use and manufacture tools was once thought to be a unique characteristic of humans^1^; however, we now know that tool-use behaviors can be observed across various taxa,^2^ including birds,^3^^,^^4^^,^^5^ fish,^6^ and dolphins.^7^^,^^8^ Nevertheless, primates stand out as the most prolific tool users in the animal kingdom, e.g., chimpanzees (*Pan troglodytes*),^9^ orangutans (*Pongo pygmaeus*),^10^^,^^11^ robust capuchins (*Sapajus* sp.),^12^ bearded capuchins (*Cebus libidinosus*),^13^ and long-tailed macaques of the Andaman Sea region of mainland Southeast Asia (*Macaca fascicularis aurea*).^14^ Humans, however, are the most technological primate of all, and the use of tools and technology permeates every aspect of our daily lives.

Despite the importance of technology for our species, the evolutionary origins of tool use remain poorly understood. In part, this is due to the remarkable difference in tool-using propensities between our closest living relatives, chimpanzees and bonobos.^15^ Together, these two species offer the closest glimpse into the behavior of our last common ancestor. Chimpanzees are second only to humans in their tool use^2^^,^^16^ and mainly use tools in the context of extractive foraging.^15^^,^^16^^,^^17^^,^^18^ By comparison, wild bonobos rarely use tools,^15^^,^^19^ and when they do use tools, it is mainly for social purposes and personal care.^15^^,^^20^^,^^21^ Thus, there appears to be something fundamentally different in the tool-use propensities of these two species, though whether this can be explained at the ecological, social, or individual level remains unclear.^19^

The tool-use repertoire of wild bonobos is limited to 13 behaviors described to date.^15^^,^^20^^,^^21^ Unlike chimpanzees, wild bonobos do not use tools in extractive foraging on a population level, although rare observations of individuals using a leaf sponge or fruit husk to drink water have been reported (Lomako^22^ and LuiKotale^23^). However, despite being rare in wild bonobos, tool use is widespread in captivity. Captive bonobos in zoos and sanctuaries exhibit a large and diverse tool-use repertoire,^24^ including the use of sticks for exploration, reaching food or objects, and extractive foraging.^24^^,^^25^ Past experimental research has demonstrated that captive bonobos are skilled tool users,^26^ show understanding of the functional properties of tools,^24^^,^^25^^,^^27^^,^^28^^,^^29^^,^^30^ and even anticipate future tool use.^31^^,^^32^ Research conducted on the language-trained bonobo Kanzi illustrates the extent of the tool-use capacities that bonobos can attain, including spontaneous stone tool knapping, the use of lexigrams to request tools from humans, and the transportation of tools to anticipated future use sites.^33^^,^^34^^,^^35^ The captive-wild contrast in bonobo tool use suggests that individual, social, or ecological factors may play a role as driving forces in tool use. To date, despite considerable interest in bonobo tool use,^15^^,^^19^^,^^20^^,^^21^ these factors have not yet been tested in a tool-using population of bonobos.

Demographic and developmental factors can contribute to an individual’s propensity for tool use (e.g., age, sex, and rearing history). Previous studies have demonstrated that tool-use behaviors, particularly complex forms, gradually develop with age. For example, robust capuchin monkeys require several years to master nut cracking,^36^ and young chimpanzees only acquire termite fishing^37^^,^^38^ and ant dipping^39^ after years of practice. Another factor that influences tool-use behavior is sex. Previous research has documented female-biased tool use in captive bonobos,^24^ potentially correlating with their status and priority of access to valuable resources.^40^^,^^41^ Demographic and developmental factors are best understood as operating in concert with social status and differential access to learning opportunities in shaping variation in tool-use propensities.

Rearing history may also play a crucial, yet underappreciated, role in tool use development. Research on chimpanzees has highlighted the importance of mothers during development,^42^^,^^43^ including for the acquisition of tool-use skills. Thus, mother-reared individuals who grow up in species-typical social groups may benefit from frequent social learning opportunities from both mothers and conspecifics, supporting the development of group-typical tool use. In contrast, other rearing histories, such as human rearing in sanctuaries, lack species-appropriate role models but may benefit from exposure to human tool use.^44^^,^^45^^,^^46^ Hence, this leads to the hypothesis that variation in early-life experience may lead to substantial individual differences in tool use.

Social factors, such as learning opportunities, may also play an important role in the acquisition of tool use skills.^47^ Social learning can result from increased interaction with a location because a conspecific is present there (i.e., local enhancement^48^), from the presence of artifacts (i.e., stimulus enhancement^49^), or from directly observing a model use tools (i.e., observational social learning^50^^,^^51^), or a combination of these. Access to tool use sites and materials can be mediated by social factors, such as the presence of tolerant role models^52^ and social status.^53^ In bonobo society, adult females and their dependent offspring have priority access to resources,^40^^,^^54^ which can be enforced through aggression by dominant individuals.^53^ Hence, dominance status may modulate the access individuals have to available social learning opportunities. However, the potential role of bonobo social status in relation to access to tool-use learning opportunities remains to be investigated.

In addition to individual and social factors, ecological factors may also contribute to patterns of tool use. Previous work has investigated the role of ecology in explaining the differences in tool use between wild chimpanzees and bonobos.^55^ For instance, ecological conditions are known to shape the emergence and expression of tool use by determining both the availability of resources that can be exploited with tools and the materials required to manufacture tools.^19^^,^^47^ Despite the fact that wild bonobos had similar access to *Macrotermes* mounds and army ants as chimpanzees, wild bonobos did not exhibit extractive foraging in termite-fishing or ant-dipping.^55^ Hence, ecological opportunities did not seem to explain the species difference in foraging tool use. However, the role of environmental factors in explaining the captive-wild dichotomy in bonobo tool use remains to be investigated.

### The current study

We examined the individual (i.e., age, sex, and rearing history), social (i.e., past and present exposure to conspecifics using tools), and environmental (i.e., tool availability) drivers of tool use in sanctuary-living bonobos in a novel group-based extractive foraging task. Identifying the drivers of tool use in captive bonobos can provide new insights into the mechanisms underlying the tool-use dichotomy between captive and wild bonobos while, more broadly, shedding light on the ecological and social conditions that shaped the evolution of tool use in our own lineage.

We studied bonobos at Lola ya Bonobo sanctuary, a rehabilitation center in the Democratic Republic of Congo, which houses rescued wild-born orphans of the illegal bushmeat and pet trade, as well as sanctuary-born mother-reared individuals. Unlike their wild counterparts, bonobos at Lola ya Bonobo exhibit an extensive and diverse tool-use repertoire, which includes a total of 28 tool-use behaviors, such as dragging branches, as well as extractive foraging behaviors, such as nut cracking.^24^^,^^56^ However, the bonobos at Lola ya Bonobo have never been observed using stick tools for extractive foraging. Previous studies have reported stick tool use in other captive bonobo groups,^24^^,^^25^^,^^57^ but none have examined the drivers of individual variation in tool use due to limited sample sizes. Lola ya Bonobo not only houses the largest population of captive bonobos but also allows for naturalistic experiments while keeping the environmental and social conditions comparable to those of wild bonobos.

**Figure 1:**
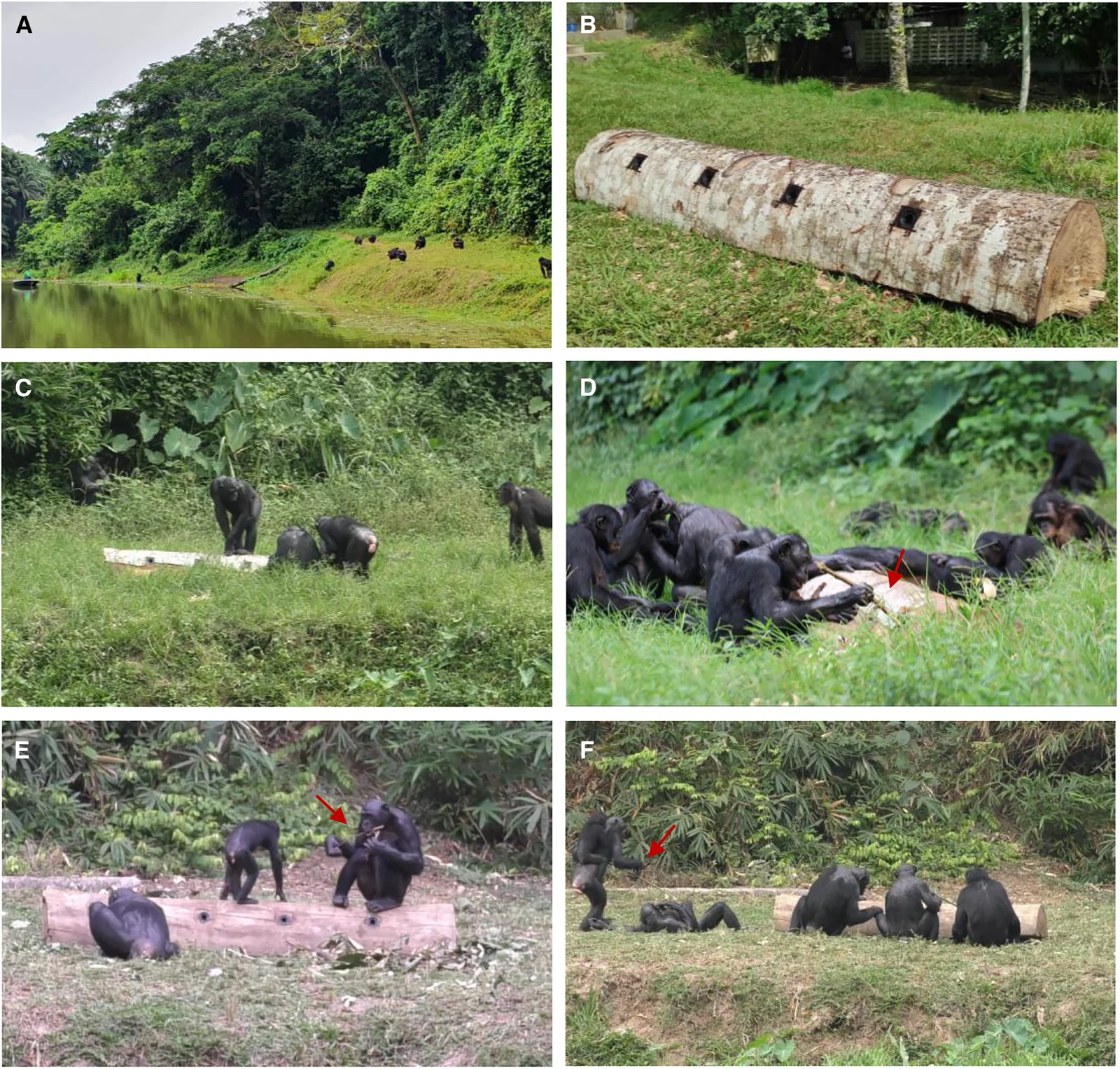
Details of the study design (A) Forested outdoor habitat at Lola ya Bonobo. (B) Experimental apparatus (section of a log). (C) Bonobos participating in the experiment. (D) Bonobo using a stick tool for extractive foraging. (E) Bonobo manufacturing a tool for use in extractive foraging. (F) Bonobo arriving at the experimental apparatus with a tool. Photo credits: Lara Zanutto/Lola ya Bonobo.

We presented two groups of sanctuary bonobos with an experimental apparatus involving an extractive foraging task that was novel to them: a large wooden log with holes containing food rewards. By providing a tool-assisted foraging task to individuals naive to the apparatus, we were able to document the relative roles of the different factors driving the acquisition and use of tools in this novel foraging task. The log was placed within each group’s 10–15 ha outdoor forested habitats such that all individuals could engage with the experimental setup (Figure 1). We quantified participation to assess socially mediated access to the tool-use site and motivation for tool use. Participation was defined as an individual’s presence within 1 m of the log. Then, for participating individuals, we recorded their tool-assisted extractive foraging to assess the individuals’ propensity for tool use.

To address the individual factors driving tool-use behavior, we first examined the effects of age, sex, and rearing history on the propensity to manufacture and use tools. We predicted that adult females and their dependent offspring would participate more frequently and exhibit more tool use than adult males. If exposure to species-relevant tool-using models during early development plays a key role in acquiring tool-using skills, we predicted that mother-reared immature individuals would show higher participation rates and greater tool use than human-reared immatures. Such differences would reflect more access to the experimental device, as well as more social learning opportunities. Second, to address the social factors driving tool use, we examined whether party size, as well as current and past exposure to tool use at the log, influenced the bonobos’ participation and tool use in the experiment. Third, to address the role of ecological opportunities, we provisioned tools at the log in an incremental way and predicted that provisioning tools would increase participation and tool use in the experiment.

## Results

**Figure 2:**
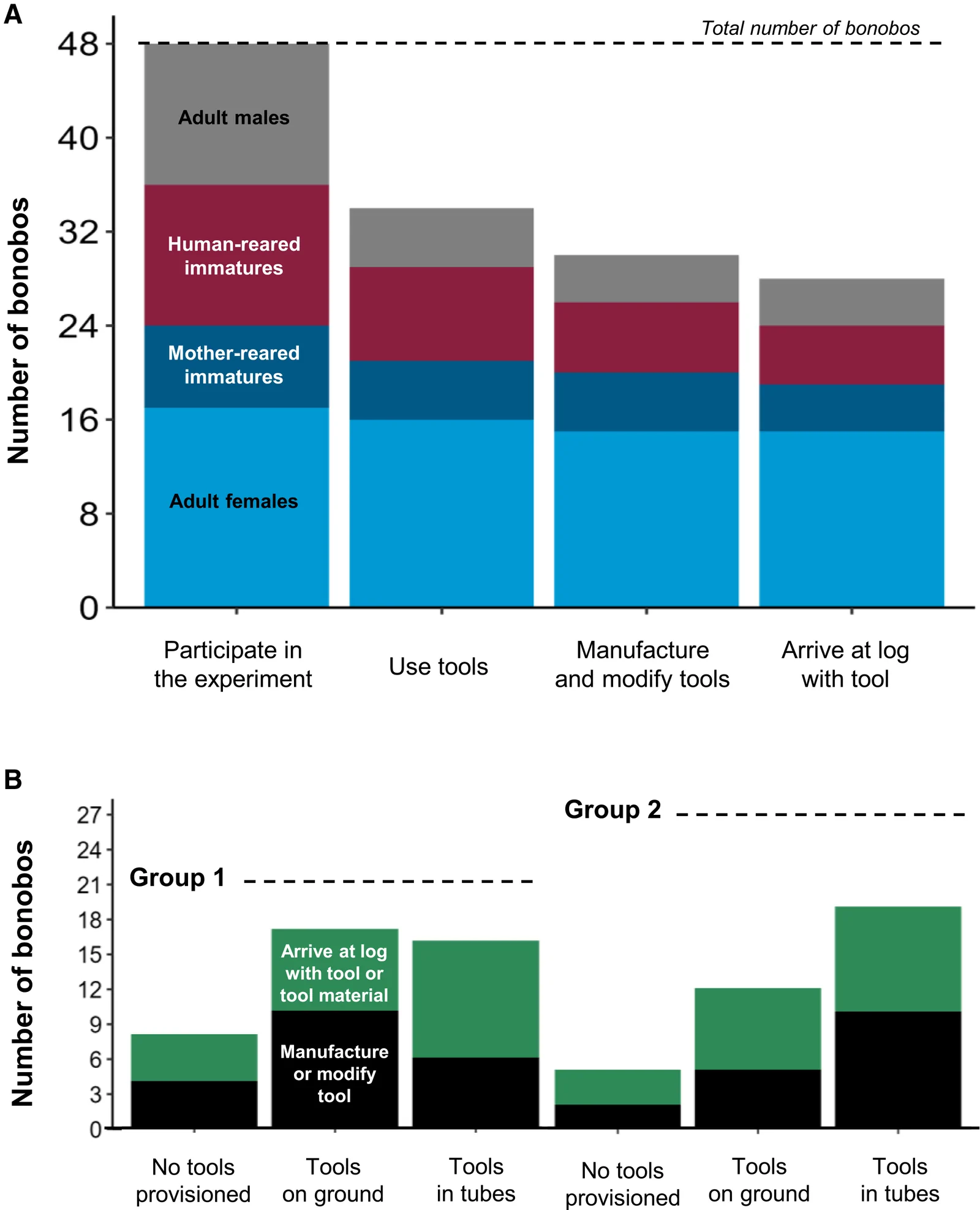
Bonobo participation in the experiment and their tool-related behaviors (A) Effect of age, sex, and rearing history on the bonobos’ type of involvement with the experiment: participation (i.e., presence within 1 m of the log, regardless of behavior), tool use, tool manufacturing or modification, and arriving at the log with a tool or suitable tool material. (B) Effect of tool provisioning on the number of bonobos observed to arrive at the log with a tool or tool material and on the number of bonobos observed to manufacture or modify tools during the experiment.

We scored experiment participation and tool use in two groups of bonobos at Lola ya Bonobo sanctuary (*N* = 48, males = 20, females = 28, 12.5 ± 8.1 years old). All engagement with the experimental apparatus was recorded during the first 3 h of exposure across 9 test days. During the days of exposure to the log, bonobos could freely choose to participate in the experiment (i.e., come within 1 m of the experimental apparatus at least once). During 51 h of footage across 9 days in Group 1 and 8 days in Group 2, we observed that *N* = 34 bonobos used tools and *N* = 30 bonobos manufactured or modified tools. Of those observed to manufacture and modify tools, *N* = 28 bonobos (58% of population) arrived at the experimental device carrying tools or materials suitable for tool use at least once (*N* = 94 observations), demonstrating evidence of tool-use planning. Furthermore, we observed a general trend that suggests adult females engaged in tool use and planned their tool use more often than males (Figure 2A). We observed that individuals manufactured and modified their tools as well as planned tool use by arriving at the log with tools or suitable tool materials with increasing frequency across the course of the experiment (Figure 2B).

**Figure 3:**
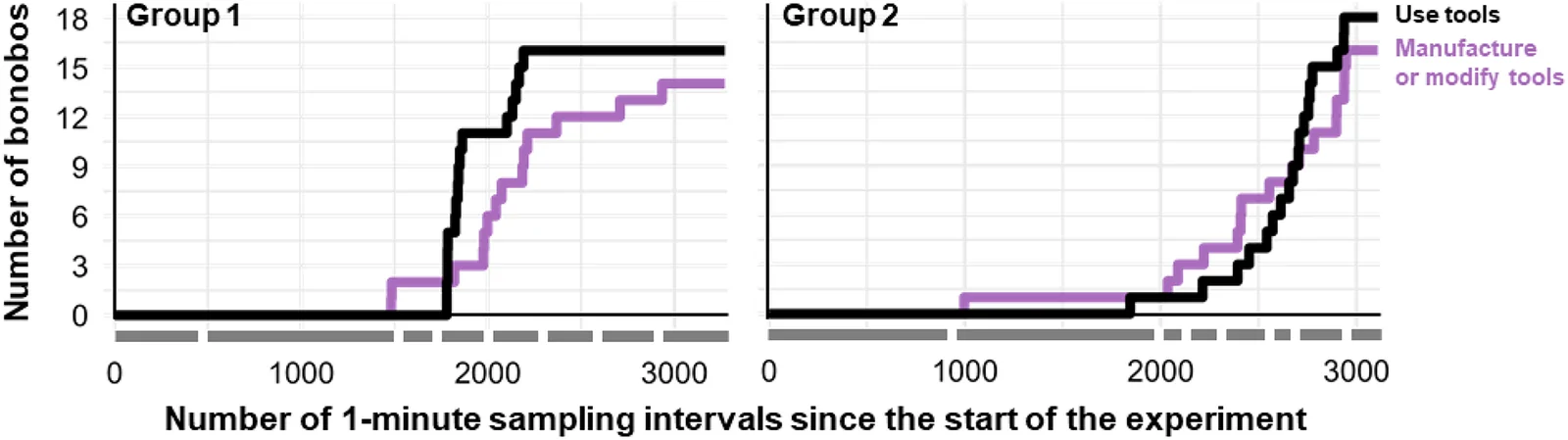
Number of bonobos who used and manufactured tools over the course of the experiment Total number of bonobos observed to engage in tool use (black lines) and to manufacture tools or arrive at the log with tools already manufactured (purple lines) across the duration of the experiment (represented as time from the start of the experiment) when bonobos were present at the log. Gray bars across the horizontal axis denote the number of days since the start of the experiment. Gray bar length varies across days because it reflects the number of sampling intervals during which bonobos were present at the log that day, rather than the total observation time.

During Period 1 (May), when no tools were provided, we observed unsuccessful tool use (i.e., tool use attempts) before we observed successful tool-use events (i.e., tool-use innovation). Within our 1-min sampling intervals, the first tool-use attempt was observed in Group 2, while the first tool-use innovation was observed in Group 1. In both groups, the first tool-use attempt observed in our sampling intervals was performed by adult females: on the second experimental day in Group 2 and on the fourth experimental day in Group 1. Tool-use innovation appeared earlier in Group 1 and spread more rapidly in Group 1 than in Group 2 (Figure 3). Specifically, in Group 1, the first observed tool-use attempt within our 1-min sampling intervals was made by Salonga, an adult female, in the early morning of the fourth experimental day. Just 1 min later, both Salonga and Opala, another adult female, achieved the first successful tool use. By the end of that day, and for the rest of Period 1, 11 individuals of Group 1, corresponding to 55% of Group 1, had been observed successfully performing extractive foraging: seven adult females (Salonga, Opala, Bandundu, Elikya, Moseka, Waka, and Katako), two adult males (Matadi and Kikwit), one juvenile female (Lutula), and a 3-year-old female (Boko), daughter of Bandundu, one of the adult females who had performed tool use. In Group 2, the first tool-use attempt within our 1-min sampling intervals was made by Boma, an adult female, in the early morning of the second experimental day and remained the only individual observed doing so that day. The second individual in Group 2 to perform extractive foraging was Kalina, another adult female, who was observed using tools for the first time on the fourth and final experimental day of Period 1. Thus, across the entirety of Period 1, only two individuals of Group 2 (Boma and Kalina), corresponding to 7.4% of the individuals of Group 2, were observed using tools.

During Period 2 (September and October), when tools were initially placed on the ground near the log and later directly inserted into the holes, tool use was observed from the first experimental day onward across several individuals. In Group 1, a total of 16 individuals were observed using tools: the same 11 individuals who had previously used tools in Period 1, plus five more individuals—three adult females (Semendwa, Kinzia, and Minova), one adult male (Pole, son of Opala), and one juvenile male (Likunzi), son of Bandundu. In Group 2, a total of 17 individuals performed tool use during Period 2: five adult females, including the two individuals who had performed tool use during Period 1 (Boma and Kalina), as well as Malaika, Ndjili, and Nyota; two adult males (Singi and Maniema); 9 juveniles (1 mother-reared: Baraka, and 8 human-reared: Mondombe, Esake, Loto, Diyoko, Mpongo, Yuli, Lolabu, and Gombe); and a 2-year-old female (Ngituka), daughter of Malaika, one of the adult females who had performed tool use.

Within our 1-min sampling intervals, the first observed instance of tool manufacture/modification in Group 1 occurred on the fourth experimental day of Period 1, performed by Likunzi, a juvenile male, though this action was not followed by tool use. Later that same day, Waka, an adult female, was the first individual of Group 1 observed to both manufacture and modify a tool and subsequently use it. In Group 2, a first attempt at tool manufacture/modification was observed on the third experimental day of Period 1 by Molongi, a 3-year-old male, who likewise did not subsequently use the object as a tool. The first observed instance of tool manufacture/modification followed by tool use in Group 2 occurred on the fourth experimental day of Period 1, performed by Kalina, an adult female and Molongi’s mother.

In terms of tool-use planning, within our 1-min sampling intervals, Semendwa, an adult female, was the first bonobo of Group 1 observed to arrive at the log with suitable tool material, on the second experimental day of Period 1. However, she did not manufacture a tool. The first observed instance of a bonobo arriving at the log carrying a suitable tool or tool material and then performing tool use was on the fourth experimental day of Period 1 by the adult female Waka. In Group 2, Ndjili, also an adult female, was the first individual who was observed arriving at the log with suitable tool material, on the first experimental day. However, she did not manufacture a tool. The first observed instance of a bonobo in Group 2 arriving at the log carrying a suitable tool or tool material and then performing tool use did not occur until the first day experimental of Period 2 (the fifth experimental day overall). This was performed by Malaika, an adult female, who arrived with a tool and performed tool use.

### Participation

**Figure 4:**
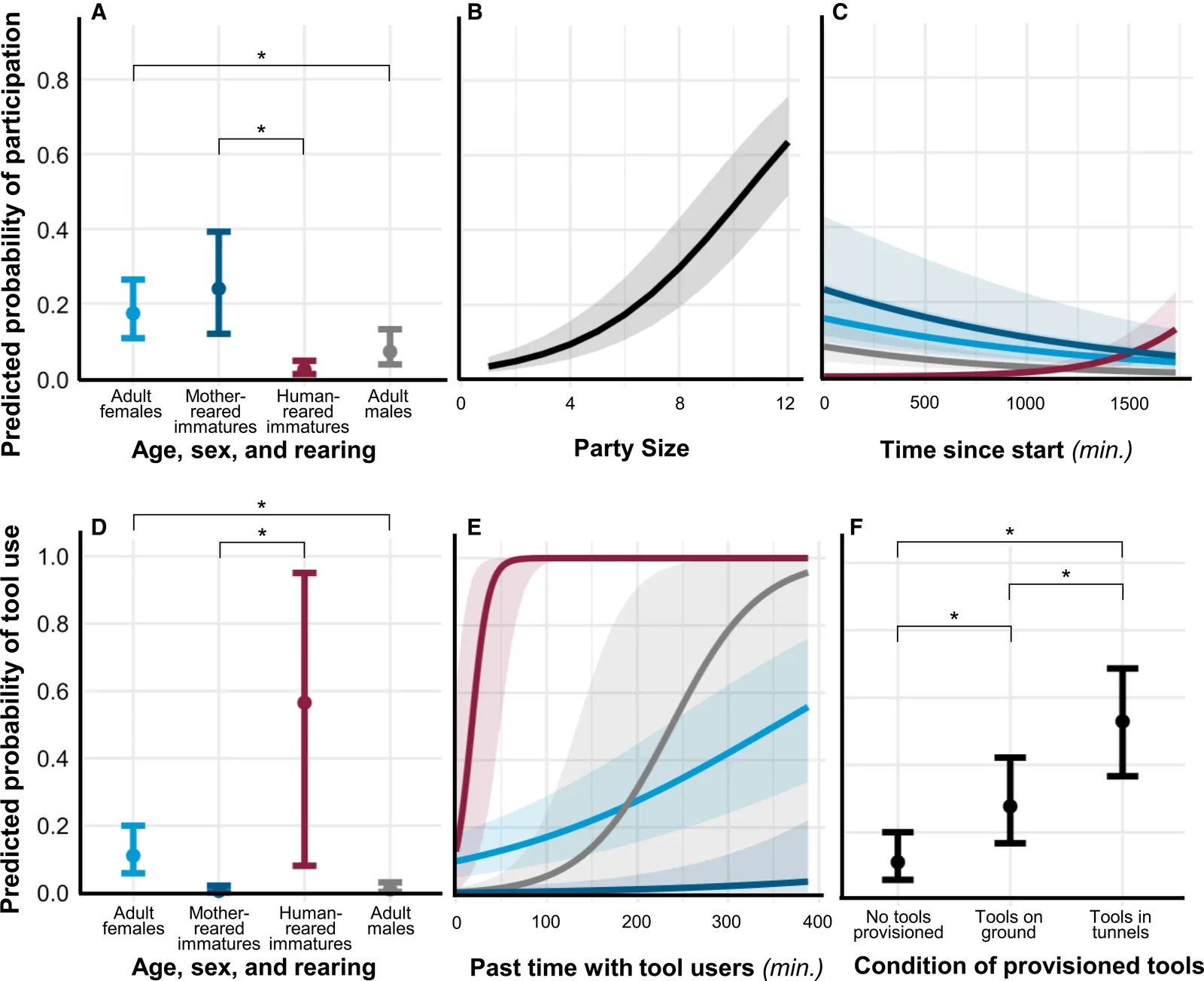
Predicted probabilities of participation and tool use in the experiment (A–C) Predicted probability of participation in the experiment across (A) age, sex, and rearing history categories, (B) in response to changes in party size, and (C) across the progression of the experiment, and a product of the interaction terms between age, sex, and rearing history. (D–F) Predicted probability of tool use in the experiment across (D) age, sex, and rearing history categories, (E) spending time in the past at the log with others who were using tools, and (F) conditions of provisioned tools. Results of both generalized linear mixed models, where predicted effects are plotted with confidence intervals (lines with caps, shaded areas). Age, sex, and rearing history categories are colored for adult females (light blue), mother-reared immatures (dark blue), human-reared immatures (red), and adult males (gray). Data are represented as model-predicted estimates (lines and points) with 95% confidence intervals (shaded bands or error bars). Asterisks, where present, indicate significant pairwise comparisons (*p* < 0.05).

Using 1-min interval sampling, we coded the presence of each bonobo within 1 m of the log during the first 3 h of each day that the log was available to assess their participation in the experiment. Each interval contributed one observation per individual, resulting in a total of *N* = 5,755 observations of bonobos at the log across all sampling intervals. The probability of participation in the experiment varied across individuals and was significantly higher for adult females than for adult males (pairwise comparison = 1.76, SE = 0.47, *Z* = 3.78, *p* < 0.001), and human-reared immatures participated significantly more than mother-reared immatures (pairwise comparison = −2.79, SE = 0.58, *Z* = −4.77, *p* < 0.001; Figure 4A).

To explore the factors that may affect participation beyond individual motivation, we examined the effect of the presence of other group members and of aggression events within 1 m of the log. Individuals were more likely to participate in the experiment in the presence of more group members, consistent with social learning through local enhancement (β = 0.35 [confidence interval (CI): 0.34, 0.37], SE = 0.01, *Z* = 56.30, *p* < 0.001; Figure 4B). In terms of aggression events, adult females exhibited aggression most often (49% acting and 31% receiving; collected via all-occurrence sampling), which is not surprising given that they frequently participated (55% of participation). Human-reared immatures received aggression more often at the log (40% of total aggression events) compared to all other age-rearing history categories (i.e., adult females, adult males, and mother-reared immatures). Receiving aggression significantly decreased the probability of participation for adult males (β = −0.40 [CI: −0.50, −0.30], SE = 0.05, *Z* = −7.90, *p* < 0.001) and immatures (human-reared, β = −0.17 [CI: −0.19, −0.15], SE = 0.01, *Z* = −15.20, *p* < 0.001; mother-reared, β = −0.11 [CI: −0.13, −0.09], SE = 0.01, *Z* = −10.08, *p* < 0.001), but not adult females (Table S1).

As the experiment progressed, the probability of participation for adult females and mother-reared immatures decreased, likely indicating a lack of interest as the novelty of the extractive foraging task diminished (Table S1). On the other hand, the probability of participation increased for human-reared immatures (β = 0.004 [CI: 0.0043, 0.0038], SE = 0.0002, *Z* = 17.30, *p* < 0.001). These results likely indicate that those who are lower status in bonobo societies (i.e., young orphaned bonobos) had greater access to the log only after adult females, who tend to be of higher status, and their offspring lost interest in the experiment (Figure 4C). Table S1 provides the complete results for the probability of participation model.

### Tool use

Across our observations at 1-min intervals during the first 3 h of each day that the log was available, we recorded 1,431 instances of tool use across all sampling intervals. The probability of tool use for extractive foraging during the experiment was significantly higher for adult females than for adult males (pairwise comparison = 1.85, SE = 0.69, *Z* = 2.70, *p* = 0.03) and for human-reared immatures compared to mother-reared immatures (pairwise comparison = 9.28, SE = 1.97, *Z* = 4.71, *p* < 0.001; Figure 4D).

To examine social factors, we tested the effect of party size, number of conspecifics using tools within one’s party (i.e., current exposure to tool use), and previous time spent at the log with conspecifics who were using tools (i.e., past exposure to tool use). Party size and number of conspecifics using tools within one’s party did not significantly influence the probability of tool use. However, previous experience with tool users (including one’s own experience with tools) produced a significantly positive effect on the predicted probability of tool use (β = 0.01 [CI: 0.008, 0.01], SE = 0.001, *Z* = 9.79, *p* < 0.001). However, this effect was stronger for human-reared immatures (β = 0.09 [CI: 0.06, 0.12], SE = 0.02, *Z* = 6.22, *p* < 0.001) and adult males (β = 0.03 [CI: 0.01, 0.05], SE = 0.02, *Z* = 6.23, *p* < 0.001) compared to adult females and their offspring (Figure 4E).

To examine the effect of ecological opportunities on tool use, we investigated the effect of provisioning tools. Provisioning tools significantly increased the predicted probability of tool use for all bonobos. Bonobos were more likely to engage in extractive foraging with tools when tools were provided, rather than not provided (pairwise comparison = −1.12, SE = 0.15, *Z* = −7.55, *p* < 0.001). The probability of tool use was higher when tools were inserted directly into the baited tubes compared to when they were placed on the ground near the log (pairwise comparison = −1.08, SE = 0.17, *Z* = −6.24, *p* < 0.001; Figure 4F). Table S2 provides the complete results for the probability of tool-use model.

## Discussion

We investigated extractive foraging tool use in the world’s largest captive bonobo population. Our results show that bonobos possess the cognitive abilities and motivation required to innovate, manufacture, modify, and use extractive foraging tools. Moreover, we showed that bonobos are capable of planning future tool use by arriving at the experimental device with pre-acquired tools or suitable tool materials. We were able to explain the inter-individual variation in bonobo tool use in relation to social, environmental, and demographic factors, shedding new light on the drivers of tool use evolution in the hominid lineage.

Variation in participation in the experiment was related to access to the experimental device, highlighting the role of social status in shaping tool use and innovation. Adult females, who occupy higher status positions in bonobo society,^58^^,^^59^ accompanied by their dependent offspring, had priority access to the experimental device, whereas adult males and human-reared immatures, who occupy lower status positions, only gained consistent access once adult females’ participation declined. This could indicate cumulative familiarization by adult females as the novelty of the extractive foraging task diminished, together with a possible learned devaluation of its effort-to-reward ratio through repeated exposure to the task. Notably, this decline occurred even as tool provisioning and individuals’ knowledge about the task increased over the study period, arguing against practical tool-access costs as the primary driver. For mother-reared immatures, declining participation more likely reflects their mothers’ reduced engagement, given the proximity typically maintained between immatures and their mothers. Adult females participated in the experiment and used tools more frequently than adult males, a pattern consistent with previous reports of female-biased tool use in captive bonobos.^24^^,^^25^ This contrasts with results from similar experiments in captive chimpanzees, where sex biases were not consistent in neither tool use nor participation.^60^^,^^61^^,^^62^ Rather than reflecting a sex difference in technical competence, the observed sex bias in bonobos may indicate differences in social status that facilitated access to the experimental apparatus. Adult males may have the same motivation to use tools as females (100% of males participated at one point or another, Figure 2), but males had limited access to the experimental apparatus likely due to their relatively low social status.

Rearing history also strongly shaped tool use in the immature bonobos in our study. We found that human-reared immature bonobos exhibited more tool use than mother-reared immatures, despite their reduced initial access to the experimental device. Although mother-reared immatures were expected to have had more social learning opportunities due to their access to maternal social learning models, they may also have benefitted more from scrounging (i.e., eating peanut butter off the end of their mother’s tools; see Figure S1) as compared to orphans. For mother-reared individuals, greater opportunities for scrounging may have led to a reduction in motivation to engage in tool use needed to obtain the resource independently. Similar patterns of scrounging influencing cultural transmission of behaviors have been documented in capuchin monkeys.^52^^,^^63^ In addition, the early-life experience of human-reared orphans in the nursery with human-provided objects and human tool use may have enhanced their motivation to engage with a tool-assisted task.^44^^,^^45^^,^^64^ Hence, rearing history and social opportunities likely act together to shape tool use acquisition in young bonobos.

The human-mediated effects in our study appear to be consistent with predictions of the enculturation hypothesis,^44^^,^^65^ which postulates that non-human apes raised by humans develop enhanced cognitive and behavioral abilities, compared to their counterparts who develop in natural environments. Our results are also relevant to the captivity effect,^64^ which posits that apes living in any sort of captive environment where there are no predators and food is provided have more free time to interact with objects and explore novel problems.^66^ Our research contributes to the view that human-mediated effects do not operate in isolation from the social and ecological factors that structure access to tool-use sites and materials.

Social learning opportunities, in terms of spending time in proximity to other individuals using tools, also influenced bonobo tool use in our study. Moreover, environmental opportunities, in terms of the provisioning of tools, played a role. The latter effect was especially strong when tools were provisioned directly into the baited tubes rather than on the ground close to the experimental device. By highlighting how social learning opportunities and the availability of tools interact with individual motivation and experience, our results provide a mechanism by which latent cognitive capacities are readily expressed in captivity. These findings inform broader models of tool use in *Pan* by highlighting that the expression of tool use may depend on the ecological and social opportunities that allow such capacities to be expressed, rather than the cognitive capacity alone. From this perspective, the rarity of tool use in wild bonobos may reflect the absence of the socio-ecological conditions required for the emergence and maintenance of tool-use traditions.

Finally, some bonobos arrived at the experimental device carrying pre-acquired tools or materials suitable for tool manufacture, rather than acquiring them opportunistically near the experimental device. The ability to plan tool use and select suitable tool materials for a future task indicates the capacity to form mental representations of suitable tools for predicted needs.^67^ These results add to the existing evidence that captive bonobos plan for future tool use^31^^,^^32^ and provide the first evidence from a semi-naturalistic captive bonobo population, bridging the gap between controlled captive experiments and conditions more closely resembling those encountered by wild bonobos.

Understanding tool use in bonobos contributes to the broader question of why habitual tool use emerges in some species and populations but not in others. Our findings suggest that the evolution of tool use in humans and other taxa was likely shaped by a similarly complex set of interacting factors. By identifying the conditions under which our closest living relatives develop tool use, we have specified the ecological and social conditions that may have supported the emergence and cultural transmission of tool use in the human lineage. In our study, bonobos successfully innovated a novel tool use behavior, highlighting the importance of opportunity in supporting the expression of latent cognitive capacities. The shared cognition necessary to express tool use in extractive foraging by bonobos and chimpanzees indicates that the key elements underlying material culture were likely present in our last common ancestor, prior to the emergence of the hominin lineage.^68^ Variation in tool use among extant apes likely reflects variation in social and ecological factors rather than fundamental cognitive discontinuities. Our results support the hypothesis that widespread tool-use abilities were a pervasive feature of early hominins, waiting for the right ecological and social circumstances to drive the emergence of diversity and complexity in our own species.^69^^,^^70^

### Limitations of the study

While this study provides insights into the drivers of tool use in captive bonobos, it does not address the ecological and social constraints that make tool-use behaviors almost absent in wild bonobo populations. It also fell outside the scope of the current investigation to explore other factors linked to captivity that may explain the difference between captive and wild bonobo tool use, such as terrestriality,^71^ neophobia,^72^ and free time.^64^ Furthermore, additional research related to the neurobiological correlates of individual variation in tool use would be of considerable interest. A comparative framework to systematically examine the ecological and social drivers of tool-use development in sanctuary compared to wild bonobos is needed to investigate how different developmental environments produce such strikingly different expressions of tool-use behaviors in this species (Zanutto et al., in prep.).

## Resource availability

### Lead contact

Requests for further information and resources should be directed to and will be fulfilled by the lead contact, Lara Zanutto (larazanutto@gmail.com).

### Materials availability

This study did not generate new unique reagents.

### Data and code availability

• Data reported in this paper will be shared by the lead contact upon request.
• This paper does not report original code. Code reported in this paper will be shared by the lead contact upon request.
• Any additional information required to reanalyze the data reported in this paper is available from the lead contact upon request.

## Acknowledgments

We thank our host Lola ya Bonobo (www.friendsofbonobos.org), Claudine André, Fanny Minesi, Raphaël Belais, and all the veterinarians, caretakers, and staff members at Lola ya Bonobo. We are very grateful to the Lola ya Bonobo staff and to Yves Nkayilu for assistance with the experiment and to Stephanie Kordon for her support in training and establishing the observational methodologies. We thank the Ministry of Research and the Ministry of Environment of the Democratic Republic of Congo for hosting our research (research permit nos. MIN.RSIT/SG-RSIT/182/180/007/2023 and MIN.RSIT/SG-RSIT/182/180/028/2023). This research was supported by the British Academy Leverhulme Small Research Grants SRG 2020–21 Round grant no. SRG2021\211185 to Z.C. and K.K. and by the 10.13039/501100001711Swiss National Science Foundation (10.13039/501100001711SNSF) grant no. PCEFP3_186967 to K.K.

## Author contributions

Conceptualization, L.Z., K.K., and Z.C.; methodology, L.Z., K.K., J.A.F., and Z.C.; investigation, L.Z., A.M., and J.A.F.; formal analysis, J.A.F. and E.P.W.; visualization, J.A.F. and L.Z.; writing – original draft, L.Z., J.A.F., and K.K.; writing – review and editing, L.Z., J.A.F., K.K., E.P.W., A.M., Z.C., and C.S.; supervision, K.K.; project administration, K.K. and Z.C.; funding acquisition, K.K. and Z.C.

## Declaration of interests

The authors declare no competing interests.

## STAR★Methods

### Key resources table

**Table:** 

REAGENT or RESOURCE	SOURCE	IDENTIFIER
**Experimental models: Organisms/strains**		

*Pan paniscus* (*N* = 48)	Lola ya Bonobo sanctuary	26 mature individuals (15 females; 11 males) and 22 immature individuals (13 females; 9 males)

**Software and algorithms**		

R	R Studio Team^73^	R: The R Project for Statistical Computing
glmmTMB package in R (version 4.3.3)	Brooks et al.^74^	CRAN: Package glmmTMB
INTERACT coding software	Mangold^75^	Mangold INTERACT – Behavioral Coding Software | macOS + Windows | Mangold

### Experimental model and study participant details

Data were collected at Lola ya Bonobo, a bonobo sanctuary accredited to the Pan African Sanctuary Alliance (PASA) located in Kinshasa, Democratic Republic of Congo, which houses the world’s largest population of captive bonobos. Most bonobos arrive at the sanctuary as orphans of the bushmeat and pet trade when they are between 6 months and 4 years old. The age of orphans is estimated upon their arrival through weight and dental development estimates.^76^ At the sanctuary, the wild-born orphans are human-reared and live in a ‘nursery’ environment together with human surrogate mothers as well as other young bonobo orphans. Bonobos remain in the nursery until approximately 5 years of age, after which they are transferred to large (10–15 ha), forested enclosures. Here, they live in mixed-age and mixed-sex groups composed of conspecifics, including both human-reared orphans and individuals born at the sanctuary and raised by their biological mothers, in adequate socially and environmentally species-typical conditions.

A total of *N* = 48 bonobos comprising the two main groups (Group 1 and Group 2) were exposed to the novel experimental task across two periods: May 2023 (Period 1) and September-October 2023 (Period 2). Due to behavioral and veterinary management reasons, group composition varied slightly depending on the experimental day. Group 1 consisted of *N* = 20 individuals (in experimental Period 1) and *N* = 21 individuals (in experimental Period 2), aged 1–29 years old. Group 2 consisted of *N* = 27 individuals, aged 1–22 years old. Individuals were classified as immatures until 8 years of age and as adults thereafter.

#### Ethics statement

This study employed non-invasive, contact-free data collection methods, and participation was completely voluntary. Beyond its scientific value, this study also promotes great ape welfare by offering enrichment opportunities.^77^^,^^78^ This research was approved ahead of time by the head veterinarian and scientific coordinator of Lola ya Bonobo sanctuary, was conducted in accordance with the Association for the Study of Animal Behavior guidelines for use of animals in behavioral research, and ethical approval was granted by the Office for Animal Welfare, Replacement, Reduction, and Refinement of the University of Zurich.

### Method details

#### Data collection

Video recordings of the experimental sessions were made using a high-definition digital video camera (Panasonic HC-VX870K 4K Ultra HD). The experiment involved the presentation of a naturalistic device with baited tubes requiring a stick tool to extract the food, thus somewhat simulating the conditions of chimpanzee ant- or termite-fishing tool use.^17^^,^^18^^,^^79^

#### Experimental apparatus

The experiment entailed the use of half of a longitudinal section of a log with holes containing a food reward (Figure 1). The log was placed in the enclosures in areas where the bonobos were commonly fed by the caregivers and where it could be safely and clearly observed from a distance. The log was obtained from an African corkwood tree (*Musanga cecropioides*) present in the sanctuary and, therefore, a familiar tree species to the bonobos. For logistical reasons, two different logs with similar measurements were used interchangeably. The logs were 2–2.25 m long and had a diameter of 30–35 cm. In each log, four holes were drilled at a distance of ∼40 cm. Each hole was designed to be fitted with a cylindric tube, which was subsequently screwed to the log with four 4-cm-long screws. This design prevented the bonobos from removing or displacing the screws or the tubes. The tubes were made of metal and had a length of 30 cm and a diameter of 3 cm. These dimensions ensured that the bonobos could not use their fingers to extract the food reward, which was placed within the last 8–10 cm at bottom of each tube. The food reward was locally sourced peanut butter. Vegetable oil was added to the peanut butter to obtain a mixture with a more fluid consistency.

#### Experimental procedure

The experiment was carried out during two data collection periods in May (Period 1) and September-October 2023 (Period 2). In each experimental period, the log was presented to each bonobo group for eight days: four in May and four in September-October 2023. However, an extra day of exposure to the experimental apparatus was added for Group 1 to compensate for an incomplete day of data collection that occurred early in the study when a bonobo pushed the log into the nearby pond after only 1 h of exposure, thus rendering the experimental device unavailable for the rest of the day. This difference of nine days for Group 1 and eight days for Group 2 was accounted for in the analyses.

During each session, the log was placed in the outdoor enclosure in the morning of the first observation day before the bonobos transferred from the indoor night enclosure into the outdoor enclosure, between 6:00 and 9:00. Once the log was in position, the tubes were baited with peanut butter, inserted into the holes, screwed to the log, and their inner surface close to the opening smeared with peanut butter. During the first experimental period (May 2023), tools were not provided to the bonobos. However, the bonobos were able to gather and manufacture tools from the nearby vegetation naturally present in the enclosure. During the second experimental period (September-October 2023), four stick tools long approximately 40 cm, sourced from the enclosure or nearby, were provided. The tools were positioned on the ground within 1-meter of the log during experimental days 5 and 6 for Group 2 and during days 5, 6, and 7 for Group 1. The tools were inserted into the tubes for the last two days in each group, i.e., during days 7 and 8 for Group 2, and during days 8 and 9 for Group 1. Bonobos were always also able to gather and manufacture tools from natural vegetation available in the enclosure.

Once the bonobos had entered the outdoor enclosure, the video recording started. Video footage was, whenever possible, accompanied by commentary by the operator(s) narrating the identity and activities of the bonobos. The positioning of the video camera allowed us to record video footage within a 2 m radius around the log. When no bonobo was visible within 10 m of the log, video recording was stopped. Video recording was resumed when at least one bonobo was visible within 10 m of the log. Video recordings ended once the bonobos had left the outdoor enclosure and entered the indoor enclosure, between 17:00 and 18:30 h. At this point, the tubes were unscrewed from the log, emptied, and washed. At the end of the last experimental day, and after the bonobos had entered the indoor enclosure, the log was removed from the enclosure.

#### Data scoring

A.M. and L.Z. scored the videos using INTERACT video coding software^75^ employing a scoring protocol. From continuous all-day video recordings, only the first 3 h of recording of each experimental day were coded (i.e., depletion of peanut butter). Some behaviors were scored using instantaneous scan sampling at 1-min intervals, while others were scored using continuous all-occurrences sampling.

The following events were scored at 1-min intervals, within a range of ±7.5 s of the sampling point, for the first 3 h of each experimental day:

1. Individual participation in the experiment, where participation was defined as being within 1-meter of the experimental apparatus.^60^
2. Attempt tool use, defined as bringing a detached object into contact/association within the log. However, the tool is unsuitable for successful extractive foraging.
3. Tool use, defined as employing an unattached or manipulable attached environmental object (the tool) to alter more efficiently the form, position, or condition of another object, another organism, or the user itself, when the user holds and directly manipulates the tool during or prior to use and is responsible for the proper and effective orientation of the tool.^2^

The following events of tool-related behaviors and aggressive behaviors were scored using all-occurrences sampling, for the first 3 h of each experimental day:

1. Tool transfer, defined as the change in possession of a suitable or unsuitable tool from one bonobo to another (adapted from Musgrave et al.^80^^,^^81^).
2. Tube sharing, when two or more bonobos use their own suitable or unsuitable tools in rapid, alternating succession to simultaneously engage with the same tube containing food for more than 15 s (adapted from Harrison et al.^82^).
3. Tool manufacture or modification, when a bonobo manufactures a tool from raw materials or modifies existing tools (adapted from Boesch and Boesch^17^).
4. Arriving at log with tool, when a bonobos arrives within 1-meter of the log with suitable tool(s), unsuitable tool(s), or raw material(s) to manufacture a tool (adapted from Musgrave et al.^83^).
5. Contact and non-contact aggressions, submissions (including unprovoked), and displacements (including unprovoked). Contact and non-contact aggressions are defined as physical contact between two or more bonobos in an aggressive context, aggressive threats, chases, and charges directed from one bonobo toward one or more clear recipients (adopted from Mouginot et al.^84^). Submissions (including unprovoked) occur when an individual exhibits a postural, facial, vocal, and/or displacement behavior directed toward another individual. Displacements (including unprovoked) occur when a bonobo takes over the physical space of another bonobo with or without physical contact.

Further details regarding the methods can be found in the Table S3.

### Quantification and statistical analysis

To test for the factors influencing bonobo participation in the experiment and tool use rates we conducted generalized linear mixed models (GLMMs), constructed using the glmmTMB package^74^ in R (version 4.3.3) using RStudio.^73^

#### Participation model construction

To examine variation in participation in the log experiment, we used a binomial GLMM to test for the influence of demography (17 adult females, 12 adult males, 7 mother-reared immatures, 12 human-reared immatures); tool provisioning (i.e., tools were not provided for 4 days in each group, tools were provided on the ground for 2 days in Group 2 and for 3 days in Group 1, tools were provided in the tubes for the last two days in each group); learning opportunities (running total number of observations of receiving tool transfer, tube sharing, and scrounging; mean = 4.37, sd = 12.70 events); receiving aggression (running total number of observations of receiving contact or non-contact aggression, being displaced, and submitting to others; mean = 2.83, sd = 5.84 events); party size (mean = 3.35, sd = 2.42); and total exposure (number of observation intervals since the start of the experiment, max = 1,731) on each individual’s participation in the log experiment (presence within 1-meter of the log). We included individual identity (*N* = 48) as a random factor to control for repeated contributions. Learning, aggression received, and exposure factors were z-transformed (forced onto a scale of −1 to 1) to improve the fit of the model. Each data point in this model corresponds to the presence or absence of each bonobo who had access to the log at every 1-min sampling interval (total observations = 5,755 present; 43,495 absent).

#### Tool use model construction

To examine variation in the bonobo’s tool-assisted extractive foraging in the log experiment, we used a binomial GLMM to test for the influence of demography (17 adult females, 12 adult males, 7 mother-reared immatures, 8 human-reared immatures), tool provisioning (i.e., tools were not provided for 4 days in each group, they were provided on the ground for 2 days in Group 2 and for 3 days in Group 1, tools were provided in the tubes for the last two days in each group), recent high-quality learning opportunities (running total number of observations of receiving tool transfer, tube sharing, and scrounging within continuous visit to the log; mean = 6.59, sd = 14.57 events), party size (mean = 3.35, sd = 2.42), total exposure (number of 1-min sampling intervals since the start of the experiment, max = 1,731), the number of others in the current 1-min sampling interval extractive foraging (mean = 1.07, sd = 1.10 conspecifics), and previous experience (number of previous 1-min sampling intervals present at the log with others who were extractive foraging, including oneself; mean = 57.93, sd = 85.55, max = 388) on each individual’s observation of extractive foraging in the log experiment. Each data point in this model corresponds to the tool use behaviors observed by each bonobos who was within 1-meter of the log at every 1-min sampling interval (total observations = 1,431 extractive foraging; 3,927 other behaviors). We included the bonobo’s individual identity (*N* = 48) as a random factor in these analyses. The exposure factor was z-transformed (forced onto a scale of −1 to 1) to improve the fit of the model. An individuals visit to the log was defined as their continuous presence within 1-meter, with absences of no longer than 10 min. The running total number of learning opportunities and receiving aggression restarted at 0 when a bonobo re-appeared within 1-meter of the log after being absent for more than 10 min. We assessed the fit of both models by ensuring a uniform distribution of simulated scaled residuals and estimable random effects structure. Additionally, we ensured that the explanatory power of both models was significantly improved over null models that contained only random effects as well as without interaction terms.
