# Supplementary material for: Bonobos innovate, plan, and transmit tool use to solve an extractive foraging task
Source: iScience. 2026 Sep 4;29(9):117443. doi: 10.1016/j.isci.2026.117443 (PMC13571968; doi:10.1016/j.isci.2026.117443)
Supplement: Supplementary file 1 [file mmc1.pdf]

iScience, Volume *29*

Supplemental information

Bonobos innovate, plan, and transmit tool use to solve an extractive foraging task

Lara Zanutto, Jake A. Funkhouser, Erik P. Willems, Ava Moser, Crickette Sanz, Zanna Clay, and Kathelijne Koops

Table S1. Results of model on the probability of participation in the experiment.

GLMM Factors	Estimate	95% CI	SE	Z	P
AgeSexRearing (af): am	-0.35	[-1.26, 0.57]	0.47	-0.74	0.46
AgeSexRearing (af): h	-5.98	[-7.08, -4.88]	0.56	-10.70	< 0.001
AgeSexRearing (af): m	0.61	[-0.47, 1.68]	0.55	1.11	0.27
Aggression received (z)	0.12	[0.11, 0.14]	0.01	13.10	< 0.001
Exposure to experiment (z)	-0.001	[-0.001, -0.0007]	0.0001	-10.70	< 0.001
Party size	0.35	[0.34, 0.37]	0.01	56.30	< 0.001
Provisioned tools (0)	0.24	[0.16, 0.32]	0.04	5.61	< 0.001
Aggression received * AgeSexRearing (am)	-0.40	[-0.50, -0.30]	0.05	-7.90	< 0.001
Aggression received * AgeSexRearing (h)	-0.17	[-0.19, 0.15]	0.01	-15.20	< 0.001
Aggression received * AgeSexRearing (m)	-0.11	[-0.13, -0.09]	0.01	-10.80	< 0.001
Exposure to experiment * AgeSexRearing (am)	-0.0003	[-0.001, 0.00001]	0.0001	-1.89	0.06
Exposure to experiment * AgeSexRearing (h)	0.004	[0.0043, 0.0038]	0.0002	17.30	< 0.001
Exposure to experiment * AgeSexRearing (m)	-0.0001	[-0.0003, 0.0001]	0.0001	-0.61	0.54
Individual ID		random (variance = 1.42, sd = 1.19)			

Nn_{obs} = 49,250, N_{ind} = 48; R^2_c = 0.49; R^2_m = 0.27; X^2 = 4344.2, DF = 13, P < 0.001

Note: AgeSexRearing categories abbreviated as adult female (af), adult male (am), human-reared immatures (h), and mother-reared immatures (m)

Table S2. Results of model on the probability of tool use in the experiment.

GLMM Factors	Estimate	95% CI	SE	Z	P
AgeSexRearing (af): am	-2.70	[-3.93, -1.48]	0.62	-4.33	< 0.001
AgeSexRearing (af): h	0.29	[-2.14, 2.72]	1.24	0.23	0.82
AgeSexRearing (af): m	-2.85	[-4.20, -1.51]	0.68	-4.17	< 0.001
Exposure to experiment (z)	-0.45	[-0.88, -0.02]	0.22	-2.06	0.04
Experience tool use by others to date	0.006	[0.004, 0.008]	0.001	6.75	< 0.001
Provisioned tools (0): 1	1.12	[0.83, 1.41]	0.15	7.55	< 0.001
Provisioned tools (0): 2	2.19	[1.69, 2.70]	0.26	8.54	< 0.001
Party size	0.04	[-0.007, 0.09]	0.02	1.69	0.09
Tool use by others in party	-0.06	[-0.16, 0.04	0.05	-1.13	0.26
Exposure to experiment * AgeSexRearing (am)	-0.45	[-1.04, 0.15]	0.30	-1.48	0.14
Exposure to experiment * AgeSexRearing (h)	-2.07	[-3.93, -0.20]	0.95	-2.17	0.03
Exposure to experiment * AgeSexRearing (m)	-0.10	[-0.80, 0.60]	0.36	-0.29	0.78
Experience tool use by others * AgeSexRearing (am)	0.01	[-0.002, 0.03]	0.01	1.76	0.08
Experience tool use by others * AgeSexRearing (h)	0.10	[0.07, 0.13]	0.02	6.34	< 0.001
Experience tool use by others * AgeSexRearing (m)	-0.001	[-0.006, 0.004]	0.003	-0.57	0.56
Individual ID		random (variance = 1.60, sd = 1.27)			

N_{obs} = 5,358, N_{ind} = 48; R^2_c = 0.61; R^2_m = 0.42; X^2 = 581.43, DF = 15, P < 0.001

Note: AgeSexRearing categories abbreviated as adult female (af), adult male (am), human-reared immatures (h), and mother-reared immatures (m)

Table S3. Ethograms of behaviors and details on the scoring procedure.

Behavior	Definition
	Behaviors scored at 1-minute sampling intervals
Participate in experiment	A bonobo is present within 1-meter of the experimental apparatus.
Tool use *(extractive forage successfully with stick tool)*	A bonobo engages in tool use with suitable tool to extract peanut butter from the tubes ± 7.5 seconds of sampling point. May be indicated by observing bonobo to be inserting suitable tool into the tubes and/or licking peanut butter off end of tool at exactly the 1-minute sampling interval.

	Behaviors scored on all-occurrence schedule
Tool manufacture or modification	A bonobo is observed to manufacture tool from raw materials or modify existing tools. Includes detaching raw materials from their source, removing auxiliary twigs or leaves, breaking off the end, splitting in half, etc.
Arriving at log with tool	A bonobo arrives within 1-meter of the log with suitable tool(s), unsuitable tool(s), or raw material(s) to manufacture a tool. Includes bonobo locomoting towards log while transporting tool(s)/material(s). Immature individuals may dismount from a conspecific's back upon arrival to the log while transporting their own tool(s)/material(s). Includes returning to the log after traveling away to gather raw material(s) or retrieve tool(s) that are not in the possession of another individual. Excludes individuals appearing in the camera's field of view but with tool(s)/material(s) but never being observed within 1-meter of the log. Also excludes individuals appearing with food items that could not be used as a tool or used to manufacture a tool (adopted from Musgrave et al., 2024).
Aggression	Includes *receiving physical agonism* (e.g., hitting, biting, pushing, pulling, slapping, grabbing, kicking, or jumping on), receiving *non-physical aggression* (e.g., threatening, chasing, charging), *submitting to another individual* (indicated through postural (e.g., crouching, lowering the body, bowing, presenting hindquarters), facial (e.g., bare-teeth displace or fear-grimace), vocal (e.g., high-pitched or whimper-like vocalizations) submissive signals), and *being displaced* (having ones space [within 1-meter] taken, including tool using location, with or without physical contact; includes fleeing upon arrival of another bonobo and fleeing from undirected dominance displays and displacements that occur outside the context of aggression. Aggressive contexts are commonly indicated by tense and sharp gestures, bare-teeth displace or fear-grimace facial expressions, and/or high-pitched cry and scream vocalizations. Excludes fleeing from conspecifics for any reason who are not directing any aggression towards them (e.g., undirected dominance displays) (adopted from Mouginot et al., 2024).

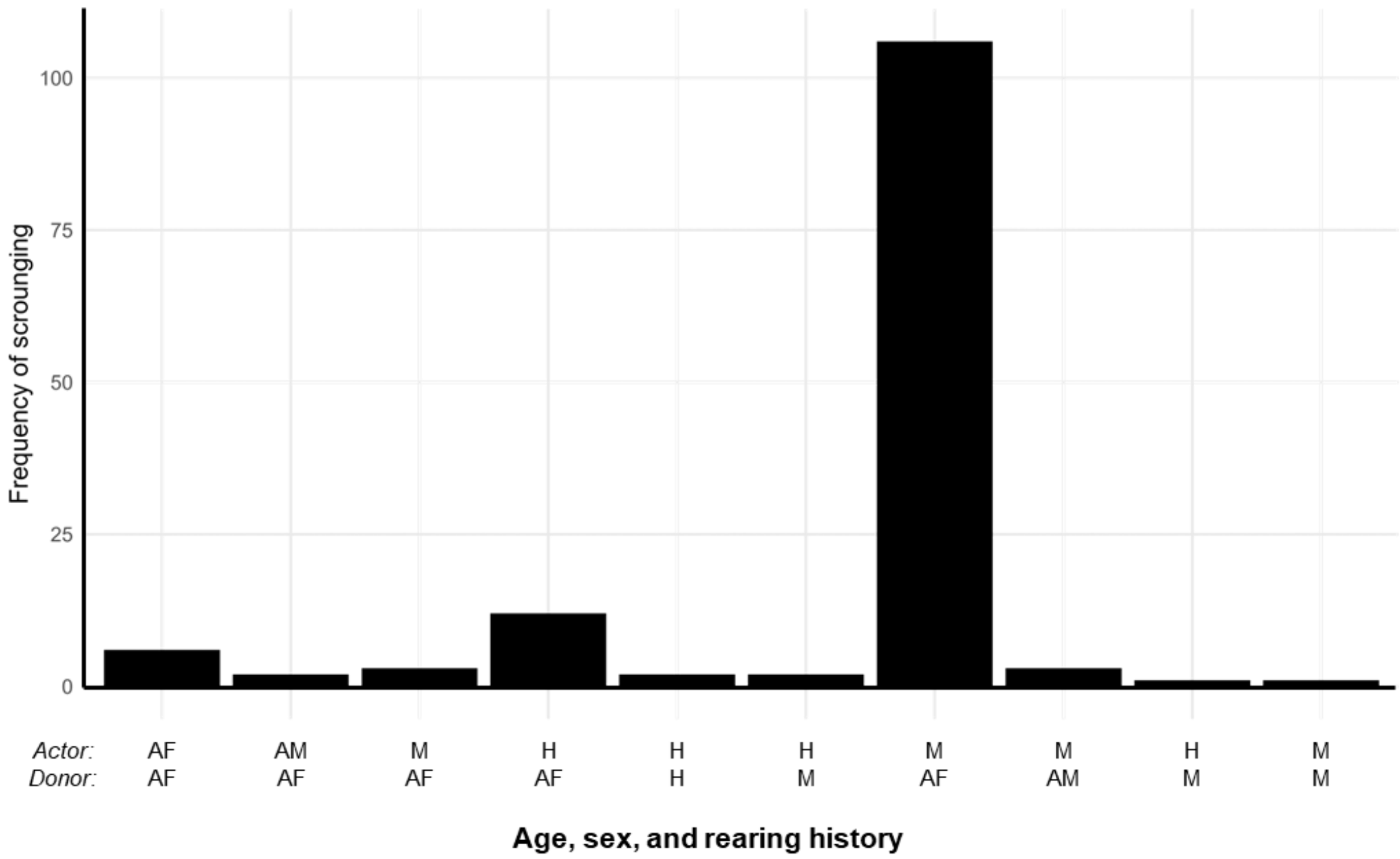

Figure S1. Observations of scrounging during the log experiment. Scrounging was defined as engaging with the products of a conspecific's tool use, most commonly eating or licking the peanut butter that was previously obtained, discarded, or partially consumed by another individual as a direct result of their tool use. These observations were not variable enough across all age, sex, and rearing history categories to an informative factor in any of our GLMM models. Age, sex, and rearing history categories are abbreviated as adult female (AF), adult male (AM), human-reared immatures (H), and mother-reared immatures (M). Across the horizontal axis, the actor of scrounging is listed first, followed by the donor/recipient (e.g., the second bar can be interpreted as the frequency of adult males observed scrounging from adult females).
